# Functional Variant rs3135500 in *NOD2* Increases the Risk of Multiple System Atrophy in a Chinese Population

**DOI:** 10.3389/fnagi.2018.00150

**Published:** 2018-05-24

**Authors:** Bei Cao, Yongping Chen, Qingqing Zhou, Lingyu Zhang, Ruwei Ou, Qianqian Wei, Ying Wu, Hui-Fang Shang

**Affiliations:** Department of Neurology, West China Hospital, Sichuan University, Chengdu, China

**Keywords:** multiple system atrophy, *NOD2*, variants, expression, association, α-synuclein, mRNA

## Abstract

**Background:** Given the overlap of clinical manifestations and pathological characteristics between Parkinson's disease (PD) and multiple system atrophy (MSA), we investigated the associations between five functional polymorphisms of nucleotide-binding oligomerization domain protein 2 (*NOD2*) which were associated with PD, and MSA in a Chinese population.

**Methods:** A cohort of 431 MSA patients and 441 unrelated healthy controls (HCs) were included in the study. Five polymorphisms in *NOD2*, including P268S, R702W, G908R, 1007fs, and rs3135500, were genotyped. The mRNA expression of *NOD2* in peripheral mononuclear cells (PBMCs) in 32 MSA patients were analyzed using RT-PCR, and the concentration of NOD2 and α-synuclein from plasma of 57 MSA patients were also measured by ELISA analysis.

**Results:** No heterozygous or homozygous for R702W, G908R, and 1007fs were found in all the subjects. For rs3135500, differences in genotype distributions, dominant and additive genetic models, were found between MSA and HCs, and between MSA Parkinsonism (MSA-P) patients and HCs. Interestingly, patients carrying the “A” allele of rs3135500 had higher mRNA *NOD2* level from PBMCs and NOD2 protein from plasma than patients without this allele (*p* = 0.028 and *p* = 0.036, respectively). In addition, we also found the concentration of NOD2 in plasma was positively correlated with the levels of *NOD2* mRNA in PBMC and α-synuclein in plasma (*R* = 0.761 and 0.832, respectively).

**Conclusion:** Our findings suggest that the rs3135500 variant in the *NOD2* gene might increase the risk for MSA and might provide new evidence that inflammation mediated by NOD2 involved in the pathogenesis of MSA. Further association studies involving a larger number of participants, as well as functional studies, are needed to confirm our current findings.

## Introduction

Multiple system atrophy (MSA) is a fatal neurodegenerative disorder resulting in autonomic failure, parkinsonism, cerebellar ataxia, and pyramidal signs. MSA has an incidence rate of three cases per 100,000 patients per year in people typically in their sixth decade of life and beyond, and is a progressive disease with a mean duration of 4.5–9.8 years from symptom onset until death (Low et al., 2015). Based on the predominant motor features, MSA is classified into two subtypes of either MSA Parkinsonism (MSA-P) or MSA Cerebellar (MSA-C) (Cykowski et al., 2015). MSA is a synucleinopathy, much like dementia with Lewy bodies (DLB) and Parkinson's disease (PD), which is characterized by the pathological presentation of widespread glial cytoplasmic inclusions (GCIs) containing α-synuclein (Cykowski et al., 2015). Multiple mechanisms, including oxidative stress, proteasomal and mitochondrial dysfunction, excitotoxicity, neuroinflammation, metabolic changes, and energy failure, are thought to contribute to the development of MSA (Jellinger, 2014). However, the underlying pathogenic mechanisms of MSA remain largely unknown.

Due to the irreversible injury of neurons in neurodegenerative diseases, great deal of researches have been devoted to explore their early diagnosis biomarkers, which might be also as the predictors of disease progression or potential therapeutic targets, or provide possible pathogenesis mechanisms for diseases, such as higher plasma levels of cystatin C in progressive supranuclear palsy (Weng et al., 2018), Alzheimer's disease and vascular dementia than that in health controls (Wang et al., 2017), which might be as a potential therapeutic mediator against neurodegeneration via VEGF-induced angiogenesis or enhanced neuronal autophagy (Zou et al., 2017). In synucleinopathes, the abnormal deposition of α-synuclein aggregates in astrocytes or oligodendrocytes has been reported to trigger inflammation (Lee et al., 2010; Vieira et al., 2015). An important role for nuclear factor κB (NF-κB)-mediated inflammation in the pathogenesis of PD and MSA has also been suggested (Schwarz et al., 1998; Hirsch and Hunot, 2009). Recently, variants including P268S, R702W, G908R, and 1007fs in the nucleotide-binding oligomerization domain protein 2 (*NOD2*) gene, encoding the NF- κB-mediated inflammation related protein NOD-2, were found to be associated with PD in Polish or Chinese patient populations (Bialecka et al., 2007; Ma et al., 2013). Additionally, a miRNA-binding variant in the 3′-UTR of the *NOD2* gene, rs3135500, which may modify miRNA-mRNA binding and alter target gene regulation, was reported to be related to another gastrointestinal tract disease in which inflammatory is an important risk factor, colorectal cancer (CRC), in Iranian (Ahangari et al., 2014). One of the current hotspot theory leading to PD is that the neuropathological process appears to start in the gut and spreads to the substantia nigra and the central nervous system (CNS) (Klingelhoefer and Reichmann, 2015), indicating that PD and gastrointestinal diseases may have a similar genetic basis.

As mentioned above, MSA shares clinical and pathologic features with PD (Stefanova et al., 2009), and chronic inflammation, such as uric acid, plays an important role in the pathogenesis of PD as well as MSA (Bialecka et al., 2007; Chen D. et al., 2015; Vieira et al., 2015). Furthermore, an accumulating body of evidence raises the possibility that MSA and PD share some genetic basis. For example, the variant rs3775444 in the *SNCA* (a-synuclein) gene, a well-established susceptibility gene in PD (Chen Y. et al., 2015), and H1 haplotype of the *MAPT* (microtubule-associated protein tau) gene, another well-known risk factor for PD (Sailer et al., 2016), have shown evidence of association with MSA. Several variants of the *LRRK2* (leucine-rich repeat kinase 2) gene, a common PD causative gene, may contribute to susceptibility to MSA (Heckman et al., 2014).

In view of the overlap of PD and MSA characteristics, the polymorphisms of *NOD2* warrant further investigation. Therefore, we performed the current study to investigate the association between P268S, R702W, G908R, 1007fs, and rs3135500 polymorphisms of the *NOD2* gene and MSA in a large cohort of Chinese population. In addition, to investigate the possible pathogenic mechanisms of candidate SNPs or NOD2 in MSA, the mRNA expression of *NOD2* in peripheral mononuclear cells (PBMCs), and the plasma NOD2 and α-synuclein were analyzed in patients carrying different genotypes of SNPs.

## Methods

### Subjects

In the current study, a total of 431 MSA patients, including 227 males and 204 females, from the Department of Neurology, West China Hospital of Sichuan University, were enrolled in the study. All patients were examined and diagnosed as probable MSA based on the Second Consensus Criteria (Gilman et al., 2008). MSA patients were categorized into two subtypes of MSA-P or MSA-C based on predominant symptoms at initial evaluation. Clinical data, including sex, age, history of smoking and drinking, age of onset, date of death and Unified MSA Rating Scale (UMSARS), were collected. A total of 441 unrelated Chinese healthy controls (HCs) (189 males and 252 females) were recruited as the control group. All the control subjects were examined by neurologists to rule out neurological disorders. Informed consent was obtained from all participants. Additionally, the study was approved by the ethics committee of the West China Hospital of Sichuan University and was conducted in accordance with the relevant guidelines.

### Genotyping

Genomic DNA was extracted from peripheral blood leukocytes using standard phenol-chloroform procedures. The frequencies of the P268S, R702W, G908R, 1007fs, and rs3135500 polymorphisms of *NOD2* were genotyped by polymerase chain reaction restriction fragment length polymorphism (PCR-RFLP). Primer sequences and restriction enzymes were used according to published studies (Ma et al., 2013; Ahangari et al., 2014). The RFLP results were confirmed by direct sequencing of the PCR products (Tsingke, Chengdu, China).

### RNA isolation and expression analysis

Total RNA was extracted from the PBMCs of 32 MSA patients (including 16 patients with the minor allele “A” of rs3135500 and 16 patients without the “A” allele) using the RNeasy Mini Kit (Qiagen, #74014). RNA purity was measured by optical density, and only samples with an OD 260/280 ratio of 1.8 to 2 and an OD 260/230 of 1.8 or greater were used. cDNA was obtained from total RNA using the QuantiTect Rev Transcription Kit (Qiagen, #205311). *NOD2* expression levels in PBMCs were measured by using a custom Real-Time PCR assay (commercial *NOD2* primer [GeneCopeia, #HQP016801]) along with the QuantiTect SYBR Green PCR Kit (Qiagen, #204143). Each real-time PCR run included within-plate triplicates and each experiment was performed twice for each sample. At the end of the PCR cycles, melting curve analyses were performed to validate the specific generation of the expected PCR product. The mRNA expression of *NOD2* was normalized to GAPDH, and the difference was calculated by the ΔΔCt method. Amplification efficiency for *NOD2* was assessed by the technical duplicates method on a Bio-Rad Real-Time PCR System.

### Enzyme-linked immunosorbent assay

After genotyping rs3135500 in all subjects, the expression of NOD2 and α-synuclein in plasma were measured in 57 MSA patients (including 4 patients with AA, 26 patients with AG and 27 patients with GG) by ELISA according to the manufacturer's instructions (Human NOD2 ELISA kit and Human α-Synuclein ELISA kit, Jijin, Shanghai, China). All NOD2 and α-synuclein in plasma were measured in duplicate and blinded for clinical phenotype.

### Statistical analysis

Polymorphisms P268S, R702W, G908R, 1007fs, and rs3135500 of *NOD2* were separately assessed for the Hardy–Weinberg equilibrium (HWE) in HCs using a Chi square test. The genotype distributions and minor allele frequencies (MAFs) of the five SNPs of *NOD2* were compared between MSA patients and HCs using Fisher's exact test. The comparison of continuous data was assessed by the Student's *t*-test. One way Anova test was performed for differences related to mRNA and NOD2 protein levels. The linear regression analysis was used to assess the relationship between plasma NOD2 and α-synuclein in patients. A two-tailed *p* < 0.05 was considered statistically significant. Statistical analysis was performed using SPSS version 23.0 (SPSS, Chicago, IL, USA). A Bonferroni correction for multiple comparisons was performed if necessary.

## Results

Demographic and clinical characteristics of the MSA patients and HCs included in the study are presented in Supplementary Table 1. There was no heterozygote or homozygote carrying minor allele for R702W, G908R, and 1007fs in the MSA patients and HCs. There were no significant deviations from Hardy–Weinberg equilibrium for P268S and rs3135500 in the HCs. No significant differences in either minor allele frequency (MAF) or genotype distributions of P268S between total MSA patients and HCs, between MSA-C patients and HCs, between MSA-P patients and HCs, or between male/female patients and matched controls were found (Tables 1, 2). For rs3135500, after adjusting for age and sex, significant differences were found in the genotype distributions (*p* = 0.04), dominant (*p* = 0.032), and additive genetic models (*p* = 0.037) between MSA and HCs. We further observed that the minor allele “A” increased the risk for MSA in females and the MSA-P subgroup (*OR* = 1.41 and 1.44, respectively) (Tables 1, 2). In addition, there were no significant differences in mean survival time and mean age of onset for MSA patients regarding rs3135500 of *NOD2* (Supplementary Table 2).

**Table 1:** Analysis of the genotype distribution, allele frequency and genetic models of variants of *NOD2* in MSA patients after adjustment for gender and age.

	**Genotype (%)**				**Allele frequencies**			**Genetic model [OR (95% CI)]**		
	**MI/MI**	**MA/MI**	**MA/MA**	***p*-value**	**MAF (%)**	***p*-value**	**OR (95% CI)**	**Dominant**	**Recessive**	**Additive**
P268S	TT	CT	CC		T					
Patients (*n* = 431)	0 (0.00)	8 (1.86)	423 (98.04)	0.198	8 (0.93)	0.199	2.22 (0.66–7.50)	2.57 (0.73–9.08)	–	–
HCs (*n* = 441)	0 (0.00)	4 (0.91)	437 (99.09)		4 (0.45)					2.57 (0.73–9.08)
rs3135500	AA	AG	GG		A					
Patients (*n* = 431)	11 (2.55)	138 (32.02)	282 (65.43)	**0.040**	160 (18.56)	0.088	1.25 (0.97–1.60)	**1.38 (1.03–1.86)**	1.26 (0.52–3.02)	1.19 (0.50–2.83)
HCs (*n* = 441)	11 (2.49)	118 (26.76)	312 (70.75)		140 (15.87)					**1.38 (1.02–1.88)**

*MI, minor allele; MA, major allele; MAF, minor allele frequency; HCs, healthy controls; OR, odds ratio. Bold values indicate significant differences*.

**Table 2:** Analysis of the genotype and minor allele distribution of NOD2 polymorphisms regarding clinical presentation in patients.

	**Genotype (%)**				**Allele frequencies**		
	**MI/MI**	**MA/MI**	**MA/MA**	***p*-value**	**MAF (%)**	***p*-value**	**OR (95% CI)**
P268S	TT	CT	CC		T		
SEX^*^							
Patients-males (*n* = 227)	0 (0.00)	4 (1.76)	223	0.999	4 (0.88)	0.999	–
HCs-males (*n* = 189)	0 (0.00)	0 (0.00)	189		0 (0.00)		
Patients-females (*n* = 204)	0 (0.00)	4 (1.96)	204	0.553	4 (0.98)	0.555	1.54 (0.37–6.44)
HCs-females (*n* = 252)	0 (0.00)	4 (1.59)	248		4 (0.79)		
**CLINICAL SUBTYPES^#^**							
MSA-C (*n* = 258)	0 (0.00)	6 (2.33)	252 (97.67)	0.086^a^	6 (1.16)	0.088	3.05 (0.85–10.94)^a^
MSA-P (*n* = 173)	0 (0.00)	2 (1.16)	171 (98.84)	0.631^b^	2 (0.58)	0.632	1.54 (0.26–9.02)^b^
HCs (*n* = 441)	0 (0.00)	4 (0.91)	437 (99.09)	0.301^c^	4 (0.45)	0.304	2.33 (0.47–11.68)^c^
Rs3135500	AA	AG	GG		A		
SEX^*^							
Patients-males (*n* = 227)	5 (2.20)	60 (26.33)	162 (71.37)	0.717	70 (15.42)	0.718	1.07 (0.73–1.57)
HCs-males (*n* = 189)	4 (2.12)	47 (24.87)	138 (73.02)		56 (14.82)		
Patients-females (*n* = 204)	6 (2.94)	78 (38.24)	120 (58.82)	0.040	90 (22.06)	**0.046**	**1.41 (1.01–1.98)**
HCs-females (*n* = 252)	7 (2.78)	71 (28.17)	174 (69.05)		85 (16.87)		
**CLINICAL SUBTYPES^#^**							
MSA-C (*n* = 258)	4 (1.55)	80 (31.01)	174 (67.44)	0.265^a^	88 (17.05)	0.455	1.12 (0.83–1.50)^a^
MSA-P (*n* = 173)	7 (4.05)	58 (33.53)	108 (62.43)	**0.017^b^**	72 (20.81)	**0.028**	**1.44 (1.04–1.98)^b^**
HCs (*n* = 441)	11 (2.49)	118 (26.76)	312 (70.75)	0.212^c^	140 (15.87)	0.228	0.81 (0.57–1.14)^c^

*MI, minor allele; MA, major allele; MAF, minor allele frequency; HCs, healthy controls; OR, odds ratio*.

* *Adjust onset age*;

# *adjust sex and onset age*.

a *Difference between MSA-C and HC*;

b *Difference between MSA-P and HC*;

c *Difference between MSA-C and MSA-P. Bold values indicate significant differences*.

**Figure 1:**
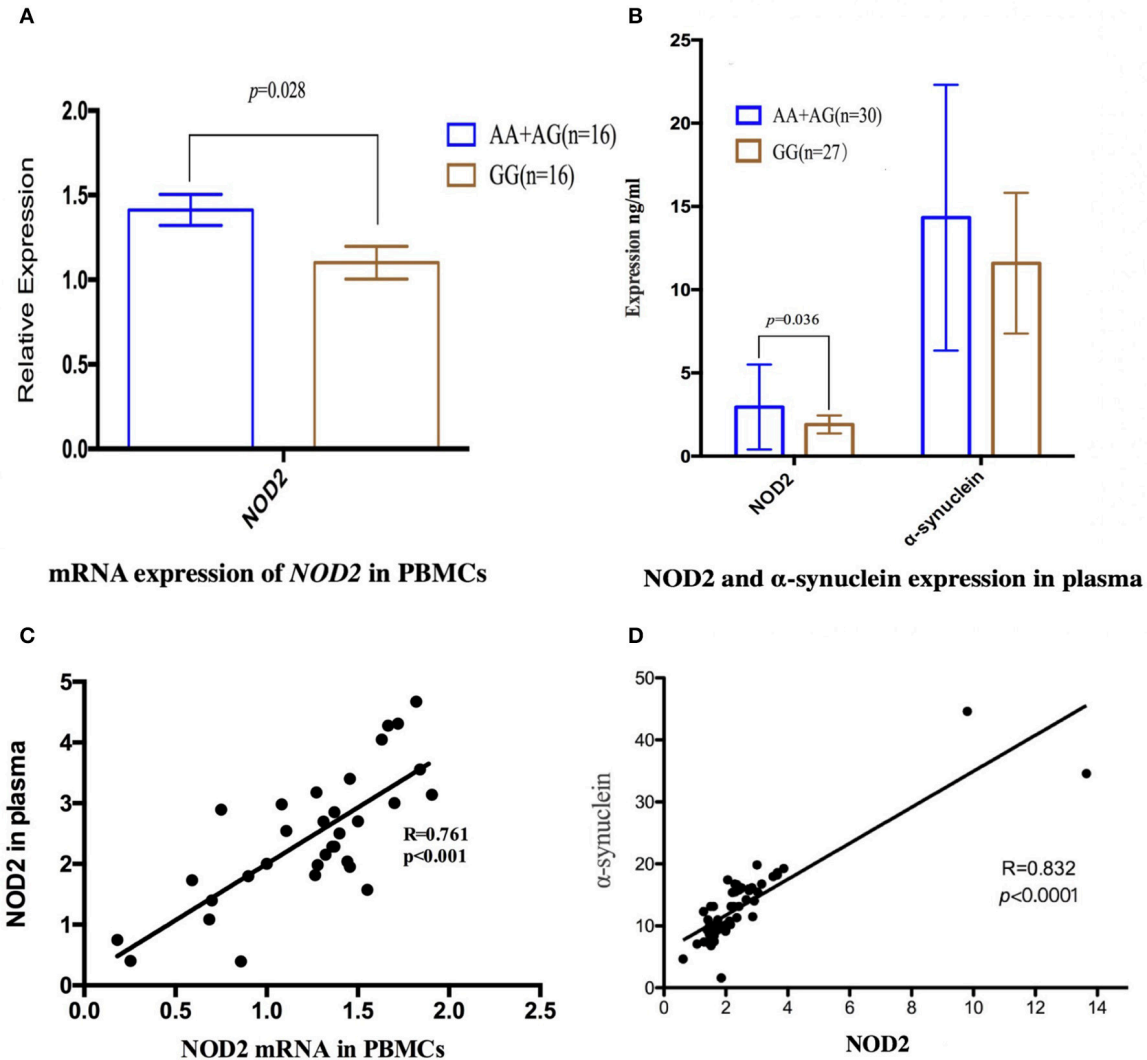
Expression of *NOD2* or α-synuclein in peripheral mononuclear cells (PBMCs) and in the plasma in the MSA patients. **(A)** mRNA expression of *NOD2* in PBMCs in MSA patients carrying minor “A” allele was significant higher than that in the patients without this allele (*p* = 0.028); **(B)** In the plasma, NOD2 protein in the patients carrying “A” was significant higher than that in the patients carrying allele “A” (*p* = 0.036); the same expression tendency was also found in the expression of α-synuclein, but there was not significant difference; **(C)** Scatterplot shows significantly positive correlation between NOD2 mRNA in the PBMCs and NOD2 in the plasma in the MSA patients (*R* = 0.761, *p* < 0.001); **(D)** Pearson correlation revealed that the expression of NOD2 was positively correlated with the expression of α-synuclein in MSA patients(*R* = 0.832, *p* < 0.0001).

To illustrate the potential pathogenesis of rs3135500 in MSA, we investigated the mRNA expression levels of *NOD2* in PBMCs and concentration of NOD2 in plasma, given that this variant is located in miRNA-binding sites. The difference in *NOD2* expression were analyzed between patients with “A” and without the “A” allele due to the limited sample size of patients carrying “AA.” Interestingly, in the PBMCs from MSA patients carrying minor “A” allele of *rs3135500, NOD2* mRNA showed a 1.28-fold increase compared to patients without the minor “A” allele (1.41 ± 0.34 vs. 1.10 ± 0.41, *p* = 0.028) (Figure 1A). In the plasma, NOD2 protein in the patients carrying the “A” allele (*n* = 30) was significant higher than in the patients without the “A” allele (*n* = 27) (*p* = 0.036) (Figure 1B). Additionally, pearson correlation analysis revealed that the levels of *NOD2* mRNA were positively correlated with the levels of NOD2 protein in the plasma in the MSA patients (*R* = 0.761, *p* < 0.001) (Figure 1C).

We also investigate the relationship between NOD2 and α-synuclein, the pathological hallmarker of MSA. Although no significant difference was found between patients with or without the “A” allele, pearson correlation analysis revealed that the expression of NOD2 was positively correlated with the expression of α-synuclein in MSA patients (*r* = 0.832, *p* < 0.001, Figure 1D).

## Discussion

This is a large cohort study of genetic susceptibility of five functional polymorphisms of *NOD2*, P268S, R702W, G908R, 1007fs, and rs3135500, in Chinese MSA patients. In the current study, we found rs3135500 increased the risk for MSA and also contribute to the variant phenotypes of MSA in a Chinese patient population (female sex and MSA-P subtype). However, we did not find the P268S, R702W, G908R, and 1007fs polymorphisms of *NOD2* modify the susceptibility to MSA or the phenotype of MSA. Through the analysis of *NOD2* mRNA and protein expression levels in PBMCs and plasma, we found the alteration of *NOD2* expression by rs3135500 of *NOD2* may be a potential pathogenic mechanism in MSA.

*NOD2*, also named *CARD15* (caspase recruitment domain family, member 15), is a member of the Nod1/Apaf-1 family and encodes a protein with two caspase recruitment (CARD) domains and six leucine-rich repeats (LRRs) (Caso et al., 2014). The protein is primarily expressed in peripheral blood leukocytes. It plays a role in the immune response to intracellular bacterial lipopolysaccharides (LPS) by recognizing their derived muramyl dipeptide (MDP) and activating the NF-κB protein (Caso et al., 2015). Mutations of *NOD2* were first described in two rare non-caseating granulomatosis diseases, Blau syndrome and early-onset sarcoidosis, which have been included in the group of NF-κB activation disorders or autoinflammatory granulomatous diseases (van Duist et al., 2005; Caso et al., 2014). Furthermore, studies focusing on the genetic background of Crohn's disease(CD) have highlighted its susceptibility in patients carrying several *NOD2* polymorphisms. For example, three SNPs of *NOD2*, 1007fs, G908R, and R702W, have been identified as susceptibility loci associated with CD in European and American populations (Inohara et al., 2003). In addition, the variant P268S was found to be associated with CD in Ashkenazi Jews (Sugimura et al., 2003) and in Chinese patients (Lv et al., 2012; Long et al., 2014). It is also interesting that a study reported that three variants (R702W, 908R, and 1007fs) of *NOD2* were significantly associated with PD in a Polish population (Bialecka et al., 2007), although this was not found in a German (Appenzeller et al., 2012) or a Chinese PD cohort (Ma et al., 2013). Meanwhile, another novel variant P268S was found to be associated with PD in a Chinese patient population (Ma et al., 2013). All of which suggested that chronic inflammation mediated by possible abnormal NOD2 by the encoding variants might be involved in the etiology of neurodegenerative diseases (Li et al., 2016). In the current study, SNP rs3135500 in the 3′-UTR of *NOD2* was found to increase the risk for MSA, which has been reported to increase the risk of CRC in an Iran study (Ahangari et al., 2014). Combination with previous study (Bialecka et al., 2007), our data may provide new evidence supporting similar pathogenic mechanisms mediated by NOD2 for both MSA and PD. In our study, we also found that this variant may modify the phenotype of MSA, since it was found to potentially increase the risk in female and MSA-P patient populations. However, additional studies with large sample sizes will be needed to confirm our current findings.

Based on a bioinformatics analysis, a previous study described rs3135500 located in miRNA-binding sites may change mRNA–miRNA interaction, resulting in dys-regulative expression of *NOD2* through targeting miR-98, miR-158, miR-215, and miR-573 (Landi et al., 2008). Further, *NOD2* was also identified as the target gene of miR-192 by luciferase reporter analysis (Chuang et al., 2014). Interestingly, we found that the mRNA level of *NOD2* in the PBMCs in MSA patients carrying “AA” or “AG” was significantly higher than that of patients carrying “GG,” which was in accordance with the principle that minor “A” suppresses the binding between mRNA and miRNA and results in a higher expression level of mRNA. Consistently, we found the patients carrying with allele “A” was related with higher plasma NOD2 compared with patients carrying with allele “G.” Therefore, in our study, we speculated a potential pathogenic role of rs3135500 in MSA is through the alteration of miRNA-mRNA binding, which could result in increased *NOD2* expression and chronic inflammation activity in the patients carrying the “A” allele of rs3135500. However, due to the limited sample size of our patients, whether the expression levels of *NOD2* in patients in the “AA” genotype was higher than that in patients in the “AG” or “GG” genotype needs to be confirmed.

Previous study found significantly elevated plasma α-synuclein levels in PD and MSA patients than in controls (Lee et al., 2006), which was concordance with our current finding. Interestingly, positive correlation between the expression of NOD2 and α-synuclein in plasma identified in the current study suggested that high or low expression level of NOD2 can reflect the level of α-synuclein. Recently, the new pathogenesis theory in PD is the spreading of pathogenesis from the periphery to the CNS (Klingelhoefer and Reichmann, 2015). Therefore, our current finding may also indicate the spreading of pathogenesis from the periphery to the CNS occurred in MSA since innate immune response mediated by NOD2, mainly occur in peripheral system, such as bone marrow immune system, gastrointestinal tract and skin, and the trigger, bacterial molecules difficultly pass the blood-brain barrier (Caruso et al., 2014). However, the causal relationship of NOD2 and α-synuclein expressions, and the concrete mechanisms of NOD2 involved in MSA need more studies.

However, in our study, we did not observe any heterozygous or homozygous carrying minor allele of R702W, G908R, and 1007fs. In addition, only 12 heterozygosity “CT” genotype of P268S were found in our cohort including 872 subjects, suggesting that these four functional variants in encoding region of the gene did not contribute to the genetic susceptibility for MSA in a Chinese patient population.

Some limitations of our study should be considered. Firstly, considering the marginally significance of rs3135500 of *NOD2* identified in MSA, further studies, including an association analysis with large sample sizes are needed. Secondly, to elucidate the role of the structural alteration of *NOD2* in this disease pathogenesis, studies including directly sequencing of all exons of *NOD2* for MSA patients are necessary.

## Conclusion

Our findings suggest that the rs3135500 variant in the *NOD2* gene might increase the risk for MSA. Our data provide preliminary evidence that inflammation mediated by NOD2 may play a functional role in the pathogenesis of MSA. Further association studies involving a larger number of participants, as well as functional studies, are needed to confirm our current findings.

## Author contributions

H-FS: planned the study; BC, YC, QZ, LZ, RO, QW, YW, and H-FS: collected and analyzed clinical data, and made patient follow-ups; BC: conducted the genetic and molecular studies, and wrote the article; YC: analyzed the data; H-FS: edited the paper.

### Conflict of interest statement

The authors declare that the research was conducted in the absence of any commercial or financial relationships that could be construed as a potential conflict of interest.
