# Supplementary material for: Functional Variant rs3135500 in NOD2 Increases the Risk of Multiple System Atrophy in a Chinese Population
Source: Front Aging Neurosci. 2018 May 24;10:150. doi: 10.3389/fnagi.2018.00150 (PMC5976778; doi:10.3389/fnagi.2018.00150)
Supplement: Supplementary file 1 [file Table_1.DOCX]

**Supplementary Table 1** Demographic and clinical characteristics of the patients and controls

| Variants | MSA | HCs |
| --- | --- | --- |
| Cases, n | 431 | 441 |
| Sex, female (%) | 204 (47.33) | 252 (57.14) |
| Mean age (mean ± SD, years) | 59.05 ± 8.62 | 56.10 ± 7.65 |
| Mean onset age (mean ± SD, years) * | 56.93 ± 8.78 | - |
| Mean disease duration (months) | 31.60 ± 19.41 |  |
| Clinical subtypes |  |  |
| MSA-C | 258 | - |
| MSA-P | 173 | - |
| Survival time^#^ (mean ± SD, months) | 60.64 ± 22.92 | - |

*Mean disease duration from onset to collection in all MSA patients;

^#^ Survival time from onset to death in 101 MSA of death;
