# Supplementary material for: Functional Variant rs3135500 in NOD2 Increases the Risk of Multiple System Atrophy in a Chinese Population
Source: Front Aging Neurosci. 2018 May 24;10:150. doi: 10.3389/fnagi.2018.00150 (PMC5976778; doi:10.3389/fnagi.2018.00150)
Supplement: Supplementary file 2 [file Table_2.DOCX]

**Supplementary Table 2** association analysis between onset age and progression and rs3135500 of NOD2

|  | Patients | Years (mean±SD) | p-value |
| --- | --- | --- | --- |
| Mean survival time |  |  |  |
| AA+AG | 42 | 4.90±1.54 | 0.477 |
| GG | 59 | 5.17±2.14 |  |
| Mean onset age |  |  |  |
| AA | 11 | 55.60±11.96 | 0.849^a^ |
| AG | 133 | 56.32±8.22 | 0.291^b^ |
| GG | 264 | 57.30±8.92 | 0.543^c^ |
| AA+AG | 144 | 56.27±8.51 | 0.257^d^ |

^a^ comparison between AA and AG; ^b^ comparison between AG and GG;

^c^ comparison between AA and GG; ^d^ comparison between AA+AG and GG
